# Pressure Sensor via Optical Detection Based on a 1D Spin Transition Coordination Polymer

**DOI:** 10.3390/s150202388

**Published:** 2015-01-22

**Authors:** Cătălin M. Jureschi, Jorge Linares, Aurelian Rotaru, Marie Hélène Ritti, Michel Parlier, Marinela M. Dîrtu, Mariusz Wolff, Yann Garcia

**Affiliations:** 1 Department of Electrical Engineering and Computer Science and Advanced Materials and Nanotechnology Laboratory (AMNOL), “Stefan cel Mare” University, University Street 13, Suceava 720229, Romania; E-Mails: catalin.jureschi@eed.usv.ro (C.M.J.); rotaru@eed.usv.ro (A.R.); 2 Laboratoire d'Ingénierie des Systèmes de Versailles, Université Versailles St Quentin, Versailles Cedex 78035, France; 3 Groupe d'Etude de la Matière Condensée (GEMaC), CNRS-UMR 8635, Université Versailles St Quentin, Versailles Cedex 78035, France; 4 ONERA - DMSC, 29 Avenue de la Division Leclerc, BP72, Chatillon Cedex 92322, France; E-Mails: marie-helene.ritti@onera.fr (M.H.R.); michel.parlier@onera.fr (M.P.); 5 Institute of Condensed Matter and Nanosciences, Molecules, Solids, Reactivity (IMCN/MOST), Université catholique de Louvain, Place L. Pasteur 1, Louvain-la-Neuve 1348, Belgium; E-Mails: marinela.dirtu@uclouvain.be (M.M.D.); mariusz.wolff@uclouvain.be (M.W.)

**Keywords:** spin crossover, pressure sensors, optical detection, smart materials, material characterization

## Abstract

We have investigated the suitability of using the 1D spin crossover coordination polymer [Fe(4-(2′-hydroxyethyl)-1,2,4-triazole)_3_]I_2_·H_2_O, known to crossover around room temperature, as a pressure sensor via optical detection using various contact pressures up to 250 MPa. A dramatic persistent colour change is observed. The experimental data, obtained by calorimetric and Mössbauer measurements, have been used for a theoretical analysis, in the framework of the Ising-like model, of the thermal and pressure induced spin state switching. The pressure (P)-temperature (T) phase diagram calculated for this compound has been used to obtain the P-T bistability region.

## 1. Introduction

**Figure 1.**
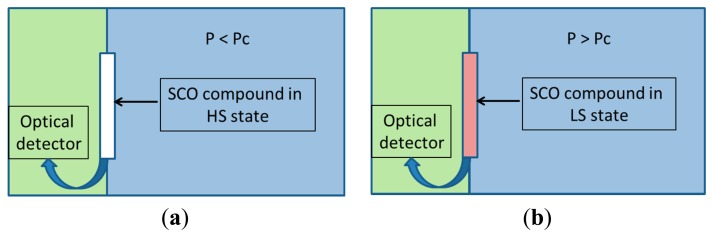
SCO material used as pressure sensor: (**a**) the SCO material reflects the light to the detector, indicating that the pressure is below the critical point; (**b**) the SCO material does not reflect the light to the detector, indicating that the pressure is above the critical point.

Due to the current need of processing and storing an ever increasing amount of information it becomes necessary to use miniaturized devices, including multifunctional components. Molecular materials are considered a particularly promising class for implementation in molecular nanodevices. To this aim, spin crossover (SCO) materials including 3d^4^-3d^7^ transition metals were selected due to their potential for miniaturization [1–5] and suitable applications in memory devices, sensors, switches, and displays [6–9]. These materials can switch between two distinguished states, namely the low-spin (LS) state and the high-spin (HS) state, by applying external perturbations such as temperature, pressure, light or a magnetic field [6]. As a result, different optical, magnetic, vibrational and structural properties are observed, which are more pronounced in the case of Fe(II) complexes. The most widely used perturbation to induce a spin state transition is the temperature, although a pressure stimulus also induces spin state modifications. Thus, SCO materials are considered as pressure sensors [7,10]. A remarkable family is represented by 1D Fe(II) coordination polymers with 1,2,4-triazole ligands [11,12], which show marked colour changes making this family of materials suitable to be used as temperature and pressure sensors with optical detection [7]. In Figure 1a, the pressure applied to the Fe(II) material is lower than the critical pressure, P_c_, and the SCO compound appears white (HS state). The white surface can reflect the laser light to the light detector, which can be connected to a computer. In Figure 1b, the pressure applied to the material under study is above the critical pressure, P_c_, causing the SCO compound to turn purple (LS state). The purple surface absorbs the light or reflects it to a lesser degree, depending on the wavelength of the light used. The change in pressure can thus be optically detected at a critical point.

The continuous developments of chemical systems in our laboratory allows the selection of the best SCO systems with special requirements concerning the SCO temperature region, which need to be located around room temperature region as well as the width of their bistability domain which need to be as large as possible. Following these conditions, we decided to focus on the 1D chain [Fe(hyetrz)_3_]I_2_·H_2_O (hyetrz = 4-(2′-hydroxyethyl)-1,2,4-triazole) as a suitable candidate. This material is known to exhibit a cooperative and thermochromic spin transition from LS (S = 0, violet) to HS (S = 2, white) around the room temperature region [13]. In this work we show that [Fe(hyetrz)_3_]I_2_·H_2_O can be used as a visual detector of strong mechanical contact pressure from 25 MPa to 250 MPa. This result is supported by thermal dependence of the optical reflectivity measurements, ^57^Fe Mössbauer spectroscopy and differential scanning calorimetry. A correlation of SCO properties with the pressure contact is made thanks to an Ising-like model. These results provide the basis for the construction of a marker pressure device.

## 2. Temperature Study

**Figure 2.**
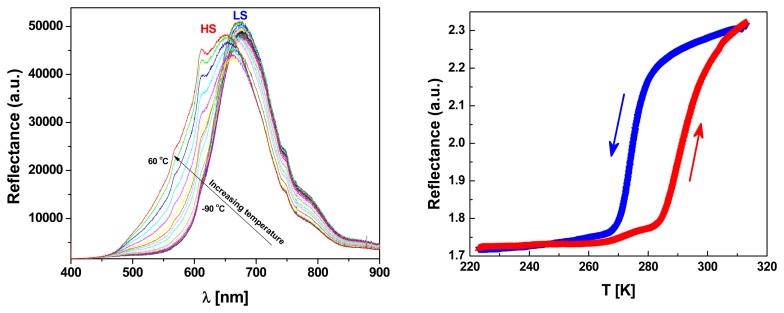
Thermal evolution of reflectance spectra recorded on [Fe(hyetrz)_3_]I_2_·I_2_O in the solid state (**left**) and of the optical density at *λ* = 550 nm (**right**).

The colour change of the sample from white (HS) to violet (LS) was monitored through variable-temperature optical reflectivity measurements in a dry nitrogen atmosphere at 2 K/min. The thermal dependence of the diffuse reflectance has been recorded, simultaneously, spectroscopically and at a quasi-monochromatic wavelength of *λ* = 550 (25) nm (see Figure 2).

The hysteretic SCO behaviour around room temperature is confirmed for [Fe(hyetrz)_3_]I_2_·I_2_O at *T_c_*^↑^ = 292 K and *T_c_*^↓^ = 275 K by optical reflectivity measurements (Figure 2). The high colour contrast of the sample, between the two spin states, can be tracked by recording temperature dependence of the reflectance spectra. Figure 2 left displays the temperature dependence of the diffuse reflectance, recorded in cooling mode. In these measurements we used a 100 W halogen bulb, which is installed on the optical microscope. At the lowest temperature, the reflectance spectrum shows a band centred on 680 nm which is assigned to the LS state, in good agreement with the pink colour of the sample. With increasing the temperature, a blue shift of the spectrum maximum is observed, followed by a broadening of the reflectance spectrum, in good agreement as well with the white colour of the sample in the HS state. It is worth noting that our reflectance spectra display an important background from the light source, which is however constant for all our experiments ensuring that the observed changes originate from the sample's colour change. By integrating these spectra using a photodiode, for each temperature, the SCO curve as a function of temperature could be obtained (Figure 2 right). The optical characterization has been completed by differential scanning calorimetric (DSC) measurements which were undertaken to confirm the spin transition temperature range as well as to determine, quantitatively, the thermodynamic parameters to be used in our Ising model described hereafter.

**Figure 3.**
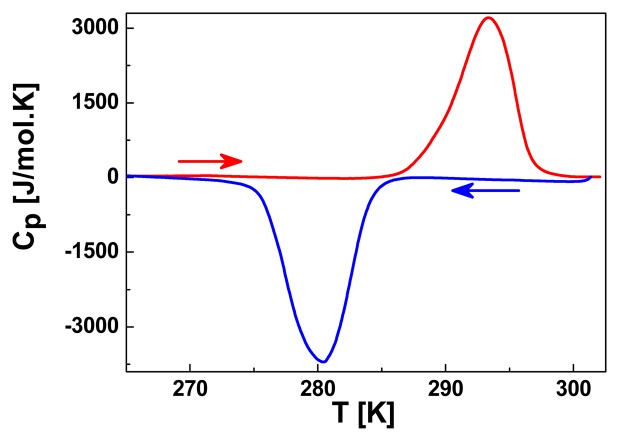
DSC curves for [Fe(hyetrz)_3_]I_2_·H_2_O over the 265–303 K temperature range.

**Figure 4.**
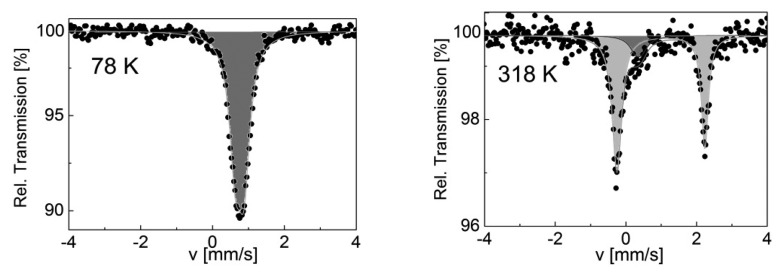
^57^Fe Mössbauer spectra for [Fe(hyetrz)_3_]I_2_·I_2_O at 78 K (**left**) and 318 K (**right**).

DSC profiles were recorded at 10 K/min in the heating and cooling modes around the room temperature region as shown in Figure 3. The DSC curve shows an endothermic peak at *T_max_*^↑^ = 293 K and an exothermic peak at *T_max_*^↓^ = 280 K, in fair agreement with the transition temperatures observed from optical measurements. The difference seen in the lower branch of the spin transition curve is associated to the lower scan rate (2 K/min) used for the optical measurements. The enthalpy and entropy variations were determined as Δ*H* = 16.42 kJ·mol^−1^ and Δ*S* = 57.42 J·mol^−1^·K^−1^. The experimentally measured entropy variation accounts for an electronic contribution, Rln5 = 13.4 J·mol^−1^·K^−1^, and a vibrational contribution of 41.77 J·mol^−1^·K^−1^. These values were corrected taking into account the active sites fraction associated with the spin transition which was accurately determined from complimentary Mössbauer spectroscopy measurements detailed below (Figure 4). Indeed on cooling to 78 K, a single quadrupole doublet with isomer shift *δ^LS^* = 0.50(1) mm·s^−1^ was observed indicating 100% LS ions. The presence of a quadrupole splitting *ΔE_Q^LS^_* = 0.24(1) mm·s^−1^, indicates a distortion of the octahedral as expected within a 1D chain [14]. At 318 K, the temperature for which the compound is expected to have undergone the spin transition, according to optical reflectivity measurements (Figure 1), the spectrum shows a major quadrupole doublet (87%) attributed to HS Fe^II^ (δ = 0.99(1) mm·s^−1^ and *ΔE_Q_* = 2.49(2) mm·s^−1^) and a minor one (13%) corresponding to LS Fe^II^ (*δ* = 0.36 mm·s^−1^ and *ΔE_Q_* = 0.17(5) mm·s^−1^). Thus, [Fe(hyetrz)_3_]I_2_·I_2_O undergoes an incomplete ST on warming with 13% of non-switching sites, which need to be taken into account for the enthalpy determination associated with the spin state change.

## 3. Pressure Study

**Figure 5.**
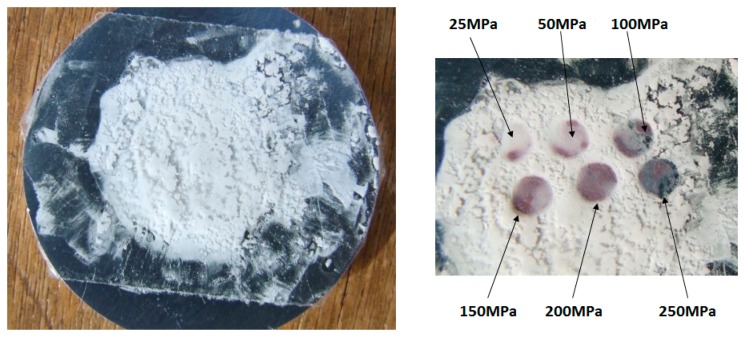
(**Left**) Sample holder showing the SCO compound at room temperature on its sample holder covered with a scotch tape; (**Right**) Enlarged view of the sample evidencing colour change to pink at room temperature for various applied pressures (25 MPa, 50 MPa, 100 MPa, 150 MPa, 200 MPa and 250 MPa).

Pressure experiments were carried out on a home-made micromechanical device. The sample was deposited on a metal plate and covered with an adhesive tape (Figure 5).

As shown in Figure 5, the material is white at room temperature and ambient pressure which is characteristic of the HS state. The pressure was applied at selected spots on the sample, released and a photograph was taken again. As it can be seen, for a threshold value of the applied pressure of ca. 30 MPa, the SCO powder switches from white (HS) to pink (LS) and retain its colour (spin state) when pressure is released. On warming to 303 K, the powder switches back to white (HS state), making the sensor reusable.

## 4. Phase Diagram Based on Ising Like Model

Various physical methods, such as magnetic susceptibility measurements, Mössbauer spectroscopy, optical and vibrational spectroscopy, X-ray diffraction and heat-capacity measurements [6], are used to describe the switching behaviour of SCO materials. Due to the expensive cost of such equipment used in physical characterizations, several theoretical models are used to predict the SCO properties. This includes models such as atom-phonon coupling [15–20], Ising-like [21–25] or mechano-elastic [16] which have been used to describe the role played in SCO behaviour by the lattice architecture, the influence of short- and long-range intermolecular interactions when applying external perturbation such as a temperature and/or a pressure variation. In this work, we have applied the Ising-like model using realistic parameters to calculate the Pressure-Temperature diagram in order to obtain the P-T bistability states. This helped us to get more information about the switching properties of the studied complex, under both temperature and pressure stimuli.

In the Ising like model, Wajnflasz and Pick [21] have introduced a fictitious spin operator *σ* to represent the two states of a SCO molecule. This operator can take the value +1 when the molecule is in the HS state or −1 when the molecule is in the LS state with respective degeneracies *g_HS_* and *g_LS_*.

For a system with non-interacting molecules the Hamiltonian is given by:

(1) $$H=\frac{\Delta }{2}\sum_{i=1}^{N}\sigma_{i}$$

where $\sum_{i=1}^{N}$ denotes the sum over all SCO molecules, *N* is the number of molecules and Δ represents the energy gap between the HS and LS states.

Over the last few years an important effort has been done in order to explain the influence of both short- and long-range interaction which induce different types of spin transition shapes [24–29], with or without hysteresis. The wideness of the hysteresis loop is determined by the long-range interaction parameter.

The Hamiltonian for interaction molecules, as proposed in [14] can be written as follows:

(2) $$H=\frac{\Delta -k_{B}T\ln g}{2}\sum_{i}\sigma_{i}-J_{i,j}\sum_{i,j}\sigma_{i}\sigma_{j}-G\langle \sigma \rangle \sum_{i}\sigma_{i}$$

where, $\sum_{i,j}$ is the sum over nearest neighbour spins, *k_B_* is the Boltzmann constant, *g* = *g_HS_* / *g_LS_* is the degeneracy ratio, *J* and *G* represent the short and long rage interaction parameters and 〈*σ*〉 is the average value of the fictitious magnetization.

In the mean field approach the average of the fictitious magnetization is given by:

(3) $$\langle \sigma \rangle =\tanh\left(\frac{2\Gamma \langle \sigma \rangle +k_{B}T\ln g-\Delta }{2k_{B}T}\right)$$

where Γ = *jq* + *G* is the reduced interaction parameter in the mean-field approximation.

The HS molar fraction is given by:

(4) $$n_{HS}=\frac{1+\langle \sigma \rangle }{2}$$

The pressure dependence of the energy gap is:

(5) $$\Delta (T,p)=\Delta_{0}+p\delta V$$

where Δ_0_ = Δ(*T*, *p* = 0), *δV* is the volume variation and *p* is the external applied pressure.

From the DSC measurements, and taking into account 87% of spin switching determined by Mössbauer measurements, the entropy value calculated Δ*S* = 57.42 J·mol^−1^·K^−1^ gives a degeneracies ratio state of the two spin state, *g*, such as ln*g* = 6.906 and the enthalpy variation give a gap energy Δ_0_/*k_B_* = 1978.6 K. For the volume variation (*δV*) during the spin transition (LS a gap energy ate, 87% oypical value of volume change *δV* = 100 A^3^.

**Figure 6.**
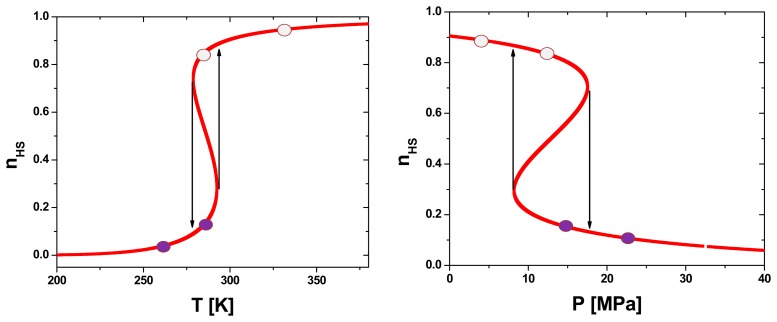
Thermal dependence at 1 bar (**left**) and pressure dependence at 300 K (**right**) of the HS molar fraction, *n_HS_*, as derived from the Ising like model. The parameters values are Δ_0_/*k_B_* = 1978.6 K, *δV* = 100 A^3^, ln*g* = 6.906 and *Γ*/*k_B_* = 360 K.

The thermal and pressure dependence of the HS molar fraction, *n_HS_*, at ambient pressure and temperature, computed for [Fe(hyetrz)_3_]I_2_·I_2_O, *i.e.*, for a complex displaying a hysteretic spin transition around room temperature is displayed in Figure 6. The transition temperatures at atmospheric pressure are *T_c_*^↑^ = 292.5 K and *T_c_*^↓^ = 278.8 K, which are in excellent agreement with the ones detected by optical reflectivity (Figure 1). The transition pressures at 300 K are *P_c_*^↓^ = 17.5 MPa and *P_c_*^↑^ = 8.2 MPa.

**Figure 7.**
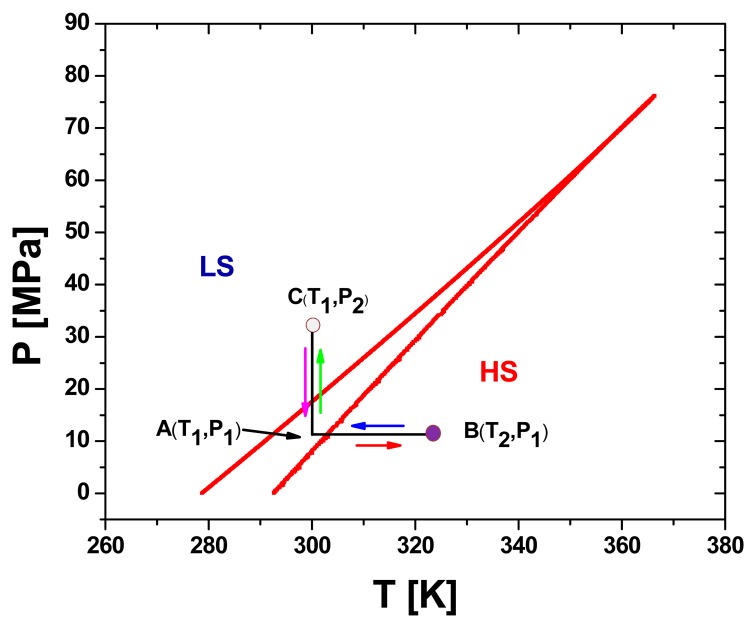
Pressure-temperature phase diagram calculated for a SCO system switching around the room temperature region using the following parameters values: Δ_0_/*k_B_* = 1978.6 K, *δV* = 100 A^3^, ln*g* = 6.906 and *Γ*/*k_B_* = 360 K.

A (P-T) phase diagram was generated to better understand the influence of temperature and pressure on the SCO behaviour. The calculated phase diagram in pressure-temperature coordinates using the same parameters as in Figure 6, is displayed in Figure 7.

**Scheme 1.**
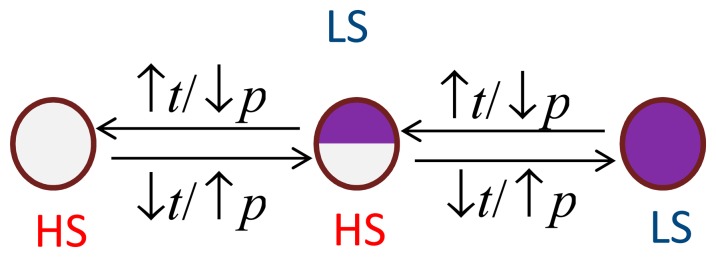
Representation of the switching mechanism induced by temperature and/or by pressure.

As can be seen from Figure 7, the transition from the HS to the LS state can be achieved either by decreasing the temperature or by increasing the pressure. When the SCO compound located at point **A**, *i.e.*, at given temperature **T_1_** and pressure **P_1_**, is heated until it reaches point **B** of temperature **T_2_** ≥ **T_up_** at a constant pressure **P_1_**, a switch back to the HS state is observed. From **B**, if the temperature is decreased until it gets back to point **A** (**T_1_**, **P_1_**) then the compound remains in the HS state. By applying a pressure, the compound switches from HS to LS states at point **C** (**T_1_**, **P_2_** > **P_down_**). If now the pressure is lowered back to **P_1_** (point **A**), the compound will remain in the LS state. In other words, inside the hysteresis width, the compound will keep the state of origin. When coming from the LS state it will remain in the LS state and when coming from the HS state it will retain the HS state as shown in Scheme 1.

## 5. Experimental

[Fe(hyetrz)_3_]I_2_·I_2_O was prepared as a solid white powder as described in [13]. The reflectance was determined by means of an optical microscope (BX51, Olympus, Center Valley, PA, USA) equipped with a photodiode and a 543 nm interferential filter, while the optical spectra have been recorded by using an UV-Vis spectrometer (Ocean Optics, Dunedin, FL, USA) mounted on the optical microscope. The sample temperature was controlled using a THMS600 liquid nitrogen cryostage (Linkam, Tadworth, UK). During the experiment the temperature was ramped at a rate of 2K/min. Differential scanning calorimetry measurements were carried out in a He_(g)_ atmosphere using a DSC Pyris 1 instrument (Perkin-Elmer, Waltham, MA, USA) equipped with a cryostat and operating down to 98 K. The purge gas was N_2(g)_. Temperatures and enthalpies were calibrated over the temperature range 98–300 K using the solid/solid and liquid/solid transitions of pure cyclopentane (P99%, Acros, Geel, Belgium) [30]. The calibration sample was introduced in an aluminum pan and hermetically sealed using an encapsulating press. The calibration was made at a scan rate of 10 K/min. The characteristic temperatures, which were assigned to the crystal/crystal transitions of cyclopentane, were obtained by the extrapolation of the onset peak temperatures. An empty aluminium pan, identical to the one used for the sample, was used as a reference to obtain a reliable baseline. The system produces or uptakes energy in order to keep the temperature of the compound identical to the reference. This energy difference between two resistances (in mW) is transformed by PYRISTM DSC Software 7.0 in specific heat *C_p_* (J·mol^−1^·K^−1^). The DSC measurement of the sample was carried out at a scan rate of 10 K·min^−1^, in warming and cooling modes. 27.7 mg were encapsulated at room temperature in aluminum pans and hermetically sealed. The sample was maintained at room temperature for 5 min in order to allow the system to equilibrate, and was further cooled down from 298 to 98 K. The sample was maintained at 98 K for 5–10 min to reach equilibrium, followed by a similar scanning mode as on cooling between 98 K and room temperature. ^57^Fe Mössbauer spectra were recorded in transmission geometry with a Mössbauer spectrometer (Wissel, Starnberg, Germany) equipped with a ^57^Co(Rh) radioactive source (Cyclotron Co., Mainz, Germany) operating at room temperature, and a Reuter-Stokes proportional counter. The samples were sealed in iron free aluminum foil and mounted on a nitrogen Oxford Instruments bath cryostat. The spectra were fitted to the sum of Lorentzian functions by a least-squares refinement using Recoil 1.05 Mössbauer Analysis Software [31]. Pressure tests were performed on a micromechanical device developed at Onera (Chatillon, France). The cylindrical indenter with a contact surface of 4 mm^2^ is fixed in the upper part and the sample holder is placed on the movable part. This latter receives the load sensor and two deformable blades are used to guide the column designed to transmit the load between the sample holder and the pressure sensor. A stepper motor is used to move the platform via a screw-nut system. A FC231100000250L load sensor (Entran, Les-Clayes-sous-Bois, France) with a capacity of 1100 N is used. During contact between indenter and sample, the moving part stops automatically when a force of 1 N is measured. For indention testing, the moving speed was 5 μ/s and the displacement is stopped when the required load is reached.

## 5. Conclusions

In this work we have proved the feasibility of pressure detection using a molecular spin crossover- based sensor/marker operating at ambient temperature. For the title compound we obtained a threshold value of the contact pressure of about 30 MPa to irreversibly induce the colour change of the molecular material, due to the spin state switching form HS to LS state. Noticeably, the possibility to switch back the colour using another stimulus (temperature) was demonstrated making this sensor reusable. The theoretical investigation in the framework of the Ising-like model allowed us to predict the bistability region for this kind of sensor applications. These results open important perspectives for molecular materials in pressure sensing applications, making possible their insertion in piezo- and thermo-chromic paints that will allow the visual detection of mechanical collisions, a very important issue in the aeronautic and automotive industries.
